# Multi-Channel Optical Coherence Elastography Using Relative and Absolute Shear-Wave Time of Flight

**DOI:** 10.1371/journal.pone.0169664

**Published:** 2017-01-20

**Authors:** Eli Elyas, Alex Grimwood, Janine T. Erler, Simon P. Robinson, Thomas R. Cox, Daniel Woods, Peter Clowes, Ramona De Luca, Franco Marinozzi, Jérémie Fromageau, Jeffrey C. Bamber

**Affiliations:** 1 CRUK Imaging Centre, Division of Radiotherapy and Imaging, Institute of Cancer Research, Sutton, Surrey, United Kingdom; 2 Joint Department of Physics, Institute of Cancer Research and Royal Marsden NHS Foundation Trust, Sutton, Surrey, United Kingdom; 3 Department of Medical Physics, Royal Surrey County Hospital, Guildford, Surrey, United Kingdom; 4 Biotech Research & Innovation Centre, University of Copenhagen, Copenhagen, Denmark; 5 Michelson Diagnostics, 1 Grays Farm Production Village, Orpington, Kent, United Kingdom; 6 Department of Mechanical and Aerospace Engineering, Sapienza University of Rome, Rome, Italy; Universite de Nantes, FRANCE

## Abstract

Elastography, the imaging of elastic properties of soft tissues, is well developed for macroscopic clinical imaging of soft tissues and can provide useful information about various pathological processes which is complementary to that provided by the original modality. Scaling down of this technique should ply the field of cellular biology with valuable information with regard to elastic properties of cells and their environment. This paper evaluates the potential to develop such a tool by modifying a commercial optical coherence tomography (OCT) device to measure the speed of shear waves propagating in a three-dimensional (3D) medium. A needle, embedded in the gel, was excited to vibrate along its long axis and the displacement as a function of time and distance from the needle associated with the resulting shear waves was detected using four M-mode images acquired simultaneously using a commercial four-channel swept-source OCT system. Shear-wave time of arrival (TOA) was detected by tracking the axial OCT-speckle motion using cross-correlation methods. Shear-wave speed was then calculated from inter-channel differences of TOA for a single burst (the relative TOA method) and compared with the shear-wave speed determined from positional differences of TOA for a single channel over multiple bursts (the absolute TOA method). For homogeneous gels the relative method provided shear-wave speed with acceptable precision and accuracy when judged against the expected linear dependence of shear modulus on gelatine concentration (R^2^ = 0.95) and ultimate resolution capabilities limited by 184μm inter-channel distance. This overall approach shows promise for its eventual provision as a research tool in cancer cell biology. Further work is required to optimize parameters such as vibration frequency, burst length and amplitude, and to assess the lateral and axial resolutions of this type of device as well as to create 3D elastograms.

## Introduction

The cellular microenvironment plays a critical role in cancer initiation, progression, and the ability to invade and metastasise [1,2]. In particular, the stiffness of the extracellular matrix (ECM) regulates cellular processes, including proliferation, alignment, adhesion, morphology, motility, lineage commitment, differentiation state, and resistance to apoptosis [3–6]. Moreover, it has recently been shown that increased local stiffness is related to the formation of the premetastatic niche [7]. Elastography, the imaging of elastic properties of soft tissues, employs an existing imaging modality to derive elasticity information by detecting displacement, strain or shear-wave speed in response to an applied stress and can provide useful information about various pathological processes which is complementary to that provided by the original modality. To date, most of the research and development in the field of elastography has been directed at clinical applications, where the requirement is for non-invasive imaging of the mechanical properties of tissues on a macroscopic scale [8]. Scaling down of elastography methods could provide invaluable information on soft tissue mechanobiology, by facilitating hitherto impossible experiments in living three dimensional (3D) cell cultures or in vivo, to study the relationship between local spatial and temporal variations in ECM stiffness and cellular behaviour. For example, experiments could be conducted to gain an improved understanding of the manner in which the mechanical properties of tumours and nearby host tissues may be associated with the invasive phenotype of cancer cells, or with cellular response to treatment.

Elastography is based upon the principle that applied stress causes sample deformation which can be measured non-invasively by existing imaging modalities and used to reconstruct the sample’s mechanical properties. There have been many proposed elastographic systems, including those based on ultrasound (US), magnetic resonance (MR) and optical imaging methods, and those that employ a dynamic, continuous, pulsed, static, unidirectional, or multidirectional applied stress. While the resolution of US [9] and MR [10] imaging is limited to about 50 μm and 120 μm respectively, OCT provides cross-sectional images of tissue anatomy, with spatial resolution on the order of 1–15 μm, to depths of about 2 mm, depicted as the spatial variation of the intensity of backscattered light [11]. OCT elastography (OCE) is a growing field of research with various techniques under development [12]. Recently OCE has been applied to detect prostate cancer ex-vivo [13], to map elasticity of freshly excised malignant breast [14], and to estimate the elastic modulus of the human ovarian tissue [15].

Most of the measurement techniques of extracellular matrix deformation require usage of implanted markers such as fluorescent beads [16–18] with subsequent correlation among the obtained images to calculate the strain. OCE, on the other hand, does not require any such additions as the information that is required for correlation is provided by the backscattering light whose origin lies in cellular inhomogeneity. In the present work we implemented a form of OCE by exciting shear waves whose local transverse displacement while propagating in the sample was in the direction of the optical axis (OCT axial) and measured their speed using OCT to track this displacement in order to detect their time of arrival (TOA) in two or more spatially separated locations. Other OCE works to date, reviewed in the discussion section of this paper, have tended to use researcher-built OCT systems designed for access to OCT signal phase information and synchronization between mechanical excitation and OCT data acquisition. Here, our main aim was to determine whether quantitative OCE would be possible by a simple user-adaptation of a commercially available OCT system, since ability to use such a system may permit broader and more rapid dissemination and application of OCE. The OCT system employed, a Michelson Diagnostics VivoSight^TM^, although was not designed to provide phase information and synchronization, is able to provide OCT A-line data acquired simultaneously from four spatially-separated OCT channels [19].

Here we report a novel 4-channel technique that was developed to take advantage of the multi-channel feature of the VivoSight™ to measure the difference in TOA of a single shear-wave burst between multiple spatial locations along the direction of propagation, which obviates the need for synchronisation between OCT data acquisition and shear wave generation. Parallel acquisition of US echo data at multiple spatial locations during the propagation of a single shear-wave pulse is a standard technique for shear-wave speed measurement in medical US using so-called supersonic shear-wave elastography (SWE) [20] and is widely available in commercial form [21]. This paper reports the first OCE implementation of an approach analogous to that normally used for US-SWE, which we call the relative TOA method.

A simple adaptation to the VivoSight™ was also made to provide a synchronization signal, permitting shear-wave speed to be determined from positional differences of TOA for any single channel over multiple shear-wave bursts, which is referred to herein as the absolute TOA method.

The aims of the work reported in this paper were two-fold: to determine whether a useful automated measure of shear-wave speed may be made using OCT amplitude-only data derived from a commercially available OCT system and to assess the novel relative TOA method of speed estimation.

## Materials and Methods

The commercial scanner employed (Michelson Diagnostics VivoSight^TM^) is a swept-source frequency-domain OCT system with a central wavelength of 1305±15 nm and total sweep range of about 150 nm. It has a unique hardware feature, in which four parallel optical-backscatter acquisition channels are used to increase the depth of focus over that of single-channel OCT systems [19] providing axial and lateral resolutions better than 10 μm and 7.5 μm respectively over a total depth range of about 2 mm. The A-line rate is 10 kHz and pixel size is 4 by 4 μm.

**Fig 1:**
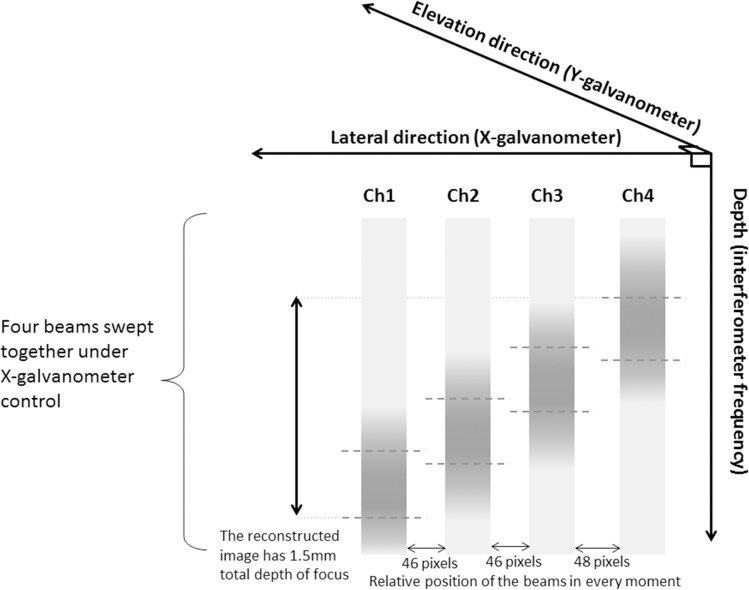
OCT system operation when acquiring a 3D stack of B-scans. At any given time after the start of a B-mode acquisition, four laser beams (each focused in different depth) are located at different lateral positions (fixed in relation to each other), where the position of the group is determined by a ramp voltage function which controls the X-galvanometer and mirror. The spatial offset between beams, depicted here in pixels, where each pixel is 4 μm, is subtracted when a B-scan is reconstructed. The focus area is indicated in dash lines and darker shades. Each B-scan is acquired at a different elevational position, determined by a voltage step which increments the Y-galvanometer and mirror.

Fig 1 shows the operation of VivoSight™ OCT hardware in which four parallel optical-backscatter acquisition channels are used to increase the depth of focus (in contrast to the single-channel OCT systems). The system acquires four A-scans in parallel which are scanned by an X-galvanometer and mirror to create a B-scan, and by a Y-galvanometer and mirror to create a 3D stack of B-scans. Each of the four channels has a laser beam focused with low numerical aperture at a specific depth, different to that of the other channels, and is used to generate a horizontal strip of backscatter data in the final image. Thus, as shown, at any given time, each beam is used to generate a section of a different A-line. In order to construct a composite B-scan image, the system applies, in software, a different lateral shift to the data selected from each channel, to compensate for the lateral spatial offsets between channels. Therefore, a reconstructed A-line consists of sections taken from four A-lines obtained at different times in the scan. If, on the other hand, the software does not compensate for the shift—as was in our experiments since we intentionally disabled this function—the A-lines are obtained at the same time, but at different spatial locations that correspond to the locations of the four channels and thus can be sued for the relative TOA method.

The four A-lines are spatially offset in the lateral direction by the distance between the laser beams. These four beams are swept laterally by an X-galvanometer and mirror, to acquire all of the A-lines to create a cross-sectional image or composite B-scan (and by a Y-galvanometer and mirror to create a 3-D stack of B-scans). Thus, at any given time, each beam is used to generate a section of a different A-line, as shown in Fig 1. In order to construct a composite B-scan image the system applies, in software, a different lateral shift to the data selected from each channel, to compensate for the lateral spatial offsets between channels. Therefore, a reconstructed A-line would consist of sections taken from four A-lines obtained at different times in the scan. The system has been used previously to create quasistatic microscopic strain images [22], and to show that propagating shear-waves can be observed and their arrival time detected manually using visual analysis of an M-mode display [23].

In the present work observation and TOA detection were automated using a cross-correlation algorithm described below, that was applied twice: a first time to estimate the time varying OCT speckle displacement in the OCT axial direction from the amplitude M-mode associated with each channel during OCT observation of a shear-wave travelling across the optical axis, which produces a trace that we call a displacement M-mode, and a second time to detect the difference in TOA of the shear wave at two spatial locations by finding the time shift that, over a given depth range, maximizes correlation between displacement M-modes obtained at those locations. In both cases a parabolic interpolation of the cross-correlation function was applied for sub-pixel estimation of the shift that maximizes correlation, as described elsewhere [24].

In order to synchronize between the needle excitation and OCT data acquisition the system was modified in the following way. The ramp voltage signal that previously controlled the X-galvanometer for lateral scanning in B-mode was disconnected (disabling lateral scanning) and instead used to trigger the generation of a shear-wave burst at the start of the display time-base for what would have been a B-scan but which, without driving the scanning mirror, became an M-scan. Furthermore, the stepped voltage signal that was previously used to increment the elevational location of the B-scans for 3D imaging was disconnected (disabling elevational scanning) and used instead to increment the lateral position of the group of four A-scan lines corresponding to the four channels. This modification allowed the acquisition of a stack of amplitude M-mode images taken at different lateral positions that were determined by the user. Within the stack, the M-scans were thus divided into groups of four, corresponding to the four channels, where between the groups the distance had been varied by lateral scanning. This terminology will be used below when describing results.

**Fig 2:**
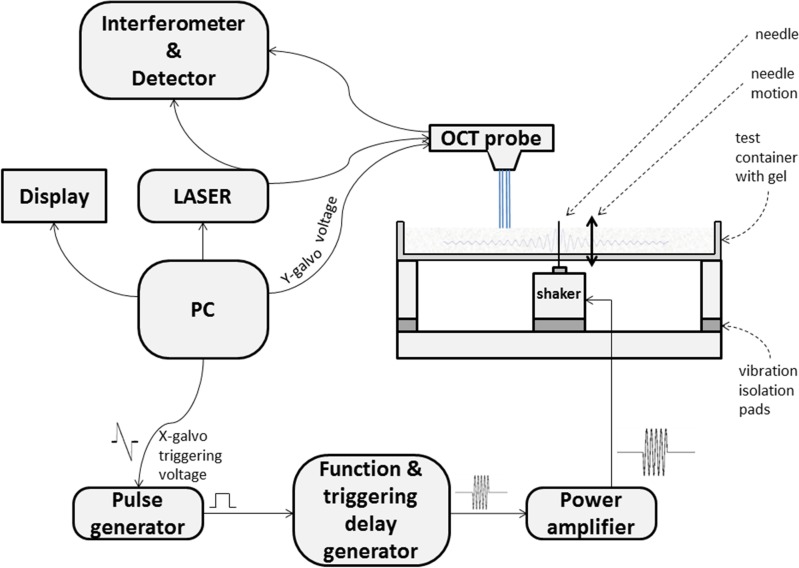
Experimental apparatus. The blue lines indicate the four laser beams and the faint grey curve a damped shear wave originating from the vibrating needle embedded in the gel (not to scale). For testing the ability to observe the passage of a shear wave in the gel, the X-galvanometer voltage cable was disconnected from the OCT probe to prevent mirror motion and lateral scanning, as described in the text.

Homogeneous gel phantoms were prepared of porcine skin gelatine (SIGMA; type A, G2625, 180 Bloom). Gelatine powder was mixed with TiO_2_ powder (T/1900/53, Fisher Scientific, UK) that was added to provide optical scatterers at a concentration of 330 mg L^-1^ by weight. The mixture was dissolved in degassed water at a temperature of 60°C and then was stirred and degassed in a vacuum for three minutes to remove all air bubbles. The suspension was allowed to cool to 27–29°C before being poured into a rectangular shallow container (210 mm in length, 10 mm in width and 5 mm in depth) to form a layer of gel 5 mm thick on a plastic (Perspex™) base, thus resembling a 3D cell culture environment. As shown in Fig 2, an audio-frequency actuator (Type 4810 Mini-shaker, Brüel & Kjær, Nærum, Denmark) provided a line-source of shear waves by vibrating a finely ridged needle set into the gel via a tiny hole in the base of the tray, similar to the approach used by Orescanin et al. for US elastography [25].

Experiments were then conducted to measure shear-wave speed in gels of varying gelatine concentration, to evaluate the reliability and the precision of shear-wave speed measurements using both the relative and absolute TOA methods described in the introduction. An expected dependence of shear-wave speed on gelatine concentration was used as a substitute for a reference medium with a known value of shear modulus, because the latter is difficult to create and because, for the range of shear moduli available with gelatine (0.25–10 kPa), the thickness of the gel layer was in the range one to six wavelengths at the frequency (500 Hz) used in this study; for such a thin layer, guided Lamb wave and Rayleigh wave propagation will occur and the wave speed will vary strongly with frequency so that the shear modulus may not be a good predictor of the expected wave speed without complex modelling of the wave propagation [26].

The wave speed should, therefore, vary nearly linearly with gelatine concentration, based on the knowledge that shear modulus varies as the square of the gelatine concentration [27]. In an unbounded medium a shear wave propagating in a lossless medium will travel with a speed, c_s_, determined by

$$c_{s}=\sqrt{\frac{G}{\rho }}$$ (1)

where G is the shear modulus and ρ is the mass density of the medium [27]. Substituting G = aC^2^ in Eq 1, where C is the gelatine concentration and *a* is a constant, gives

$$c_{s}=\sqrt{\frac{a}{\rho_{gel}}}C$$ (2)

The density of the gel should vary as ρ_gel_ = b C + √ρ_water_ [28], with the density of water acting as a large offset, modified only weakly by the concentration dependence, which itself is within the square root in Eq 2. Thus the speed of the shear wave should be nearly linearly dependent on gelatine concentration [29]. However, for a wave propagating in a thin layer, as the speed is varied by varying concentration, the ratio of wavelength to layer thickness will vary. Nevertheless, to a first approximation the near linear dependence of c_s_ on C may hold. The intention of the work was to test the hypothesis in two ways: experimentally and using finite element model (FEM).

First, FEM was used to investigate the propagation of transverse waves in a free-boundary thin layer (210 x 5 mm). A transient dynamic analysis of a two-dimensional plane strain model [30–32] was carried out with the commercial software package MARC/Mentat (MSC Software, Santa Ana, CA, USA). The medium was assumed to be isotropic, homogeneous, linear elastic and quasi-incompressible (i.e. the Poisson’s ratio was 0.49) with a density of 1000 kg/m^3^ and a Young's modulus in the range from 1 to 30 kPa, to be consistent with the values measured experimentally in gels. Damping was neglected in this study [25,32]. A needle vibrating through the thickness of the sample was modeled by exciting all the nodes on a vertical line with the same load. The excitation consisted in a sine-burst of five cycles with a central frequency 500 Hz. Temporal and spatial resolution of the model is crucial for the convergence of the numerical solution [32,33]. To avoid numerical instability and obtain an accurate solution, a suitable time step Δt was chosen according to the condition:

$$\Delta t\leq \frac{1}{20f_{max}}$$ (3)

where f_max_ is the highest frequency of interest [32,33]. In order to spatially resolve the propagating waves, the element size (L_e_) was derived from the smallest wavelength λ_min_ according to the following condition [32,33].

$$L_{e}\leq \frac{\lambda_{min}}{20}$$ (4)

A mapped and uniform mesh with squared elements [25,31,34] and an implicit solver were used.

Transverse wave displacements were gathered at several points along a horizontal line away from the needle on the top area of the plate. The speed of the transverse waves was calculated using the cross-correlation based tracking algorithm.

**Fig 3:**
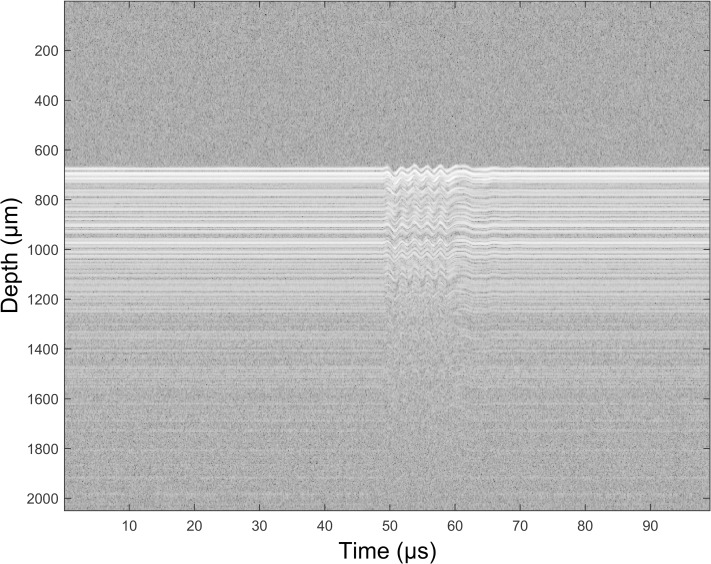
A multichannel composite M-mode scan of the phantom. Each A-line is displayed with the same (zero) lateral offset, which in M-mode is a temporal offset. However, because the four laser beams are at slightly different distances from the needle, the upper (fourth) channel data will show the shear wave arriving slightly in advance of that shown by the third channel, which will be in advance of the second etc.

Taking the experimental approach, Fig 3 depicts a single-channel OCT amplitude M-mode image at a single A-line position 1 mm from the needle in a gel of concentration 10%, during the passing of the shear wave generated by vibrating the needle with a voltage signal that was a five-cycles 500 Hz sinusoidal burst triggered when the X-galvanometer drive voltage reached a given threshold. A one-dimensional (1D) version of a cross-correlation algorithm previously described for tracking ultrasound speckle [24] was developed in the present work to detect the time varying shear-wave transverse displacement as the OCT axial shift that maximizes the correlation between a 1D reference window at a given depth in one A-line and a 1D search window in a subsequent A-line in the same M-mode.

The algorithm may be used to estimate the axial displacement of the reference window in all A-lines with respect to its axial position in a single A-line (such as the first A-line in the M-mode), which we call fixed-reference tracking. Repeated application of the algorithm for multiple depths of (overlapping) reference windows produces a 3D dataset of axial displacement as a function of depth and time. When this is displayed as an image, using displacement as image brightness, we refer to this as a displacement M-mode. Alternatively, the algorithm may be used to estimate the axial displacement of the reference window between two A-lines a fixed time apart, i.e., where the reference A-line moves in time with the A-line to be searched, which we call incremental tracking. Repeated application for all pairs of A-lines and all depths of (overlapping) reference windows once again produces a displacement M-mode dataset. Fixed-reference tracking displays true displacement but has the potential of suffering from long-term decorrelation of the speckle if there is bulk motion of the medium. Incremental tracking in effect measures the time differential of axial displacement, which is strongly dependent on the A-line separation time. Either can be used to generate a displacement-time waveform which can then be used to detect TOA of the shear wave.

For the work reported in this paper the incremental displacement tracking algorithm, implemented in software using Matlab™ (The Mathworks, Natick, MA), was used. It allows the following variables to be adjusted, each of which can affect performance: reference window size, search window size, axial skip factor, temporal skip factor and temporal interval. The axial skip factor is the distance between sequential axial positions of the centre of the reference window, which if set to a large number will reduce computation time at the expense of axial sampling interval in the displacement M-mode. The temporal skip factor is the distance between sequential A-lines upon which references windows are located, which if set to a large number will reduce computation time at the expense of temporal sampling interval in the displacement M-mode. The temporal interval is the distance in time samples between the A-line containing the reference window and the A-line containing the search window.

A study was conducted to determine optimum values for these variables, which resulted from two compromises: i) between resolution (small reference window) and low displacement noise in a homogeneous medium (large reference window), and ii) between short computation time (large skip factor and small search window) and both resolution (small skip factor) and low noise (search window large enough). The reference window chosen was 15 pixels, the search window was 30 pixels, temporal interval was one A-line and both skip factors were zero. An introduced displacement regularization method involved two steps: noise rejection followed by averaging over depth. Averaging displacement over depth sacrifices depth resolution for improvement in signal to noise ratio (SNR). The noise rejection step aims to ensure that only reliable displacement values are included in the average. For the homogeneous gels studied here, depth resolution was of no interest. Therefore, averaging of all values in each column of the displacement M-scan was carried out, destroying all depth resolution. In a future system, the trade-off could be adjusted differently, preserving some depth resolution while gaining some SNR improvement.

Three types of noise rejection were employed. First, only plausible axial shifts were accepted; that is, all displacement values that were greater than an upper displacement threshold of 1.5 pixels were rejected. The logic of this upper threshold was that the amplitude of shear wave had been adjusted so that the true inter-A-line displacement could not be greater than one axial pixel. Any displacement greater than 1.5 pixels (6 μm) was therefore judged as noise. Second, rows in the displacement M-scans that were dominated by displacement noise were removed by calculating the standard deviation of displacement over each row and rejecting rows with a standard deviation greater than a threshold value of 0.4, leaving data which tended to be centred on the depth range of good OCT amplitude SNR. This was slightly different for each channel. Third, all remaining displacement values that were less than a lower threshold were regarded as noise and were excluded from further analysis. The logic of this type of noise suppression filter is that at a time in the displacement M-scan when the true displacement signal is known to be absent any non-zero displacement must be noise. In order to find the optimal value of the lower noise suppression threshold, the depth-averaging step was carried out for a number of values of the threshold between 0.1 to 0.9 pixels displacement. From the results it was decided that a lower noise suppression threshold 0.25 provided the displacement-time waveform with the best compromise between noise reduction and preservation of a prominent sinusoidal pattern. The resulted displacement-time waveforms describe the average propagation of a shear wave over depths extending from the gel surface to a depth of about 600 μm.

Each such regularized displacement-time waveform was then processed using a second application of the cross-correlation tracking algorithm, by comparing it with a regularized waveform obtained at a different distance from the needle, either using a different OCT channel, in which case the same 5-cycle burst was used (the relative TOA method), or using the same OCT channel, in which case a different 5-cycle burst was used (the absolute TOA method). The time-shift that maximized the cross-correlation coefficient was taken as the time of shear wave propagation from one spatial location to the other. Division of the difference between arrival times by the difference between the distances from the needle of the two spatial locations, whether it were the inter-channel distance (relative TOA method) or inter-group distance (absolute TOA method), provided the measurement of shear-wave speed.

**Fig 4:**
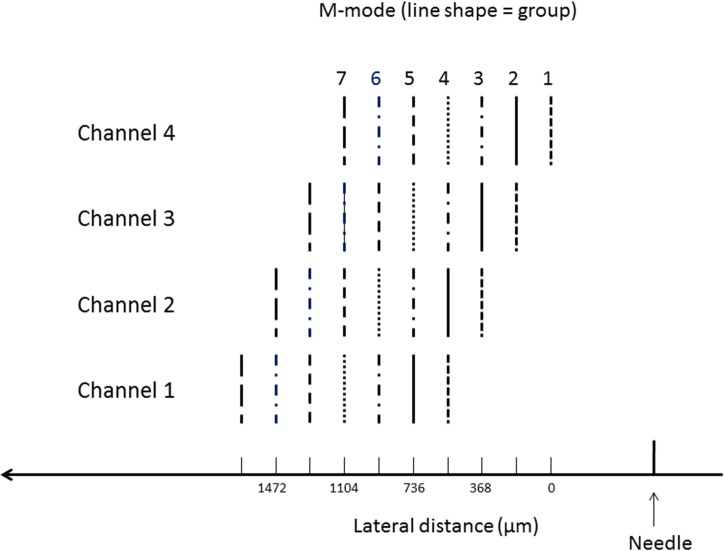
Data acquisition sequence. Each group of M-mode scans simultaneously provides information from four channels located at distinct lateral distances 184 μm apart and indicated by similar line style. The first group of such M-mode scans is acquired at an arbitrary starting distance from the vibrating needle, defined here as zero on the lateral distance axis. Subsequent groups are acquired at lateral distance increments from the needle that are equal to the inter-channel distance, which is 184 μm. These data therefore allow the comparison of measurements of TOA differences between channels at the same group position with those between group positions for the same channel.

To compare the absolute and relative TOA methods a phantom with gelatine concentration of 7.5% was prepared. The gel was scanned as described above to create seven groups of M-modes, where the distance of the first group from the needle was 0.5 mm and, to provide a fair comparison, the spatial separation between groups was equal to the distance between the channels, namely 184 μm. This scan sequence is illustrated in Fig 4, where each group of M-scans is indicated by different pattern. TOA differences for the six inter-group distance intervals were used to calculate shear-wave speed for the absolute method, allowing one such estimate for each of the six group pairs and the four channel positions, and those for the three inter-channel distance intervals were used for the relative method, allowing an estimate for each of the three channel pairs and the seven group positions. Twenty four and twenty one speed measurements, each from two TOA estimates, were therefore obtained using the absolute and relative methods, respectively. Means and standard deviations of the shear-wave speed were then calculated for each method to allow comparisons between the methods in terms of the trend of mean value and the precision of the measurements.

In addition, the data from the relative and absolute methods was further processed to study the improvement in precision of the measurement that can be achieved at the expense of lateral spatial resolution in an OCE image. This was done by using all channels to estimate the shear-wave speed rather than just two in the relative method and one specific channel in each group for all groups in the absolute method. The latter could be carried out in the same manner for other three channels. For this purpose a straight line was fit to each group of three inter-channel measurements or to one chosen channel data in the inter-group measurements by the method of least squares. Thus the total distance over which the speed was measured in the relative method (between channel 1 and channel 4) was three times that between any two sequential channels, i.e. 552μm and in absolute method it depended on the distance between the first and the last group. These methods are referred to as the 4-channel method and an absolute multi-group method respectively.

After verifying the superiority of the relative (2-channel) and 4-channel methods, the following experiments were carried out. Ten gels, two for each of a different gelatine concentration of 5%, 7.5%, 10%, 12.5% and 15% were prepared. For each gel two locations along it at a known distance from the needle were chosen as positions from which the measurements would begin. These were chosen to be 1.25 mm and 1.75 mm. For each such starting point a number of groups with varying distance between them was determined. The number of group and the distance between them were a trade-off between not being too close to a needle (to ensure that the measurement is not influenced by boundary effects at the source) and not being too far from it (to avoid a region where waves die out). Six different distances between the groups were chosen, and their number was adjusted within the aforementioned restrictions. Table 1 provides the full information.

**Table 1:** The spatial locations where M-mode data were acquired in the experiment that evaluated the automated OCE technique for shear-wave speed measurement in homogeneous gelatine phantoms.

The distance from the needle (mm)	The distance between the groups (mm)	Number of groups
1.25	0.1	13
1.25	0.15	9
1.25	0.184	7
1.25	0.2	7
1.25	0.3	5
1.25	0.5	3
1.75	0.1	21
1.75	0.15	15
1.75	0.184	13
1.75	0.2	11
1.75	0.3	7
1.75	0.5	5

In order to test whether the value of shear modulus obtained by measuring shear-wave speed is compatible with a direct measurement of shear modulus, we moulded a gelatine phantom of 15% concentration–prepared by the same protocol–to be used in compressional (DSA, TA Instruments, New Castle, USA) and torsional (AR-G2, TA Instruments, New Castle, USA) rheometers. The relation between the two is given by Eq 1, where ρ = 1.

## Results

**Fig 5:**
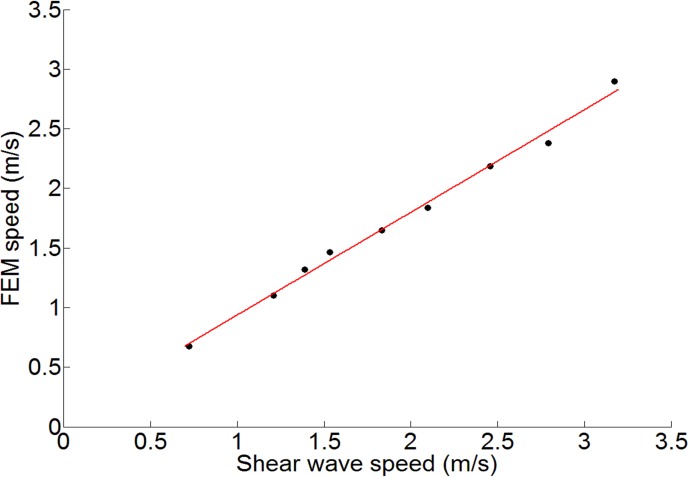
Best linear fitting of FEM transverse wave speed and shear-wave speed.

Linear regression has been used to model the relationship between the estimated speed from FEM simulations of wave propagation in the thin free layer and the shear-wave speed that would be expected for an infinite medium, where both varied as a consequence of altering the simulated gelatine concentration (Fig 5). Goodness of the fit f(x) = p_1_x + p_2_, where f(x) is the shear wave speed and x is the apparent speed, is shown in Table 2.

**Table 2:** Goodness of the linear fit of FEM apparent speed vs. shear-wave speed.

p_1_ (with 95% confidence bounds)	p_2_ (with 95% confidence bounds)	R^2^
0.8603 (0.7943, 0.9264)	0.07891 (-0.05668, 0.2145)	0.9927

**Fig 6:**
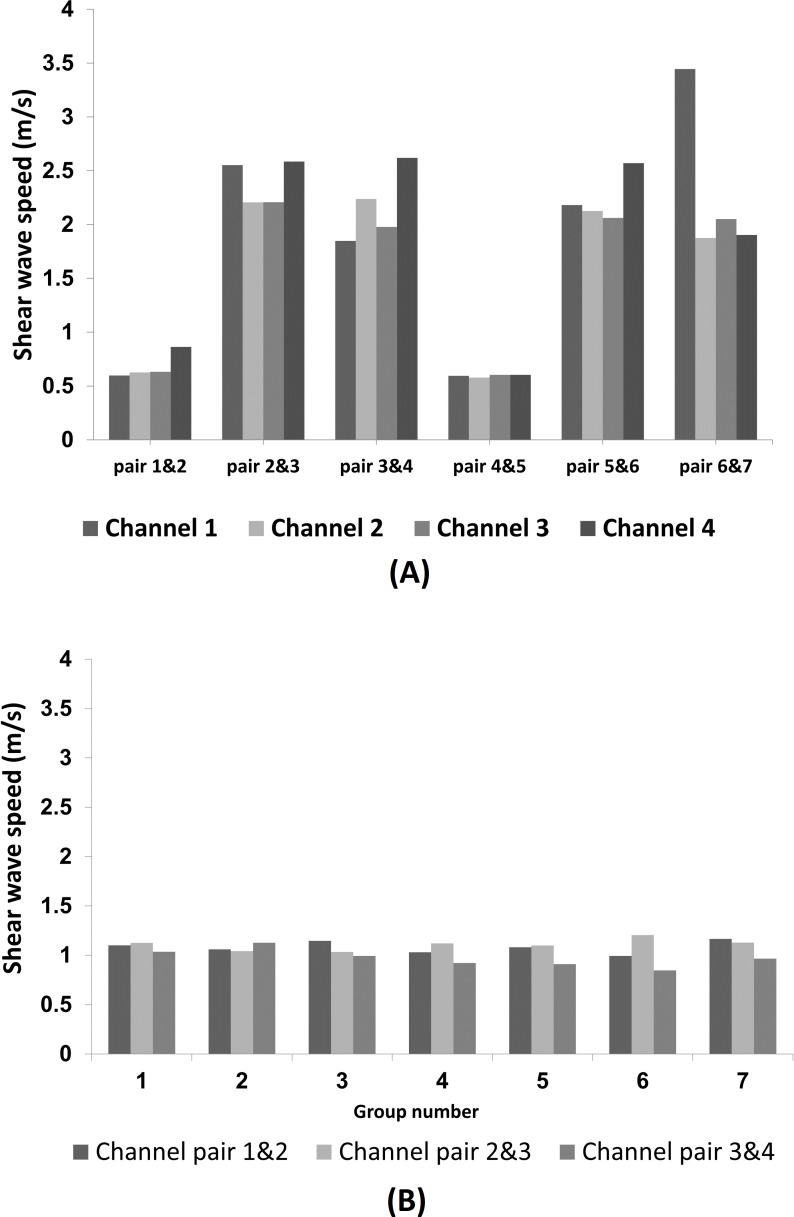
**The speed of the propagating shear wave measured for the 7.5% gelatine phantom using the absolute TOA (between-burst) method (A) and inter-channel relative TOA (within-burst) method (B).** The results represent one single experiment and therefore no standard deviation is given. Nevertheless, a mean and a standard deviation can be calculated over the six inter-group intervals for (A) and over the seven group acquisitions for (B).

As an example of the results from a single gel, those from the absolute single-group and relative 2-channel TOA methods for the 7.5% gelatine phantom, are in Fig 6A and 6B, and are summarized in Tables 3 and 4, respectively. The differences in precision between the relative (2-channel) and the 4-channel methods seen in the figures and tables were typical of those found at other gelatine concentrations. Tables 5 and 6 show the results for the aforementioned gel concentration using the relative 4-channel and absolute multi-group methods.

**Table 3:** The averaged shear-wave speed and the standard deviation (SD) over the three inter-channel measurements for each group using the relative 2-channel TOA method, for the phantom with a gelatine concentration of 7.5%. The bottom row shows the overall mean speed, the standard deviations of the means of the groups, the standard deviation over all 18 measurements (column two) and the average of the between channel-pair standard deviations (column three).

Group number	Shear-wave speed (m/s)	SD (m/s)
1	1.07	±0.05
2	1.08	±0.04
3	1.06	±0.08
4	1.02	±0.09
5	1.03	±0.10
6	1.02	±0.11
7	1.08	±0.10
Mean (± inter-group SD)	1.05±0.02	0.09
± overall SD	±0.10	

**Table 4:** The average shear-wave speed and the standard deviation (SD) over the six inter-group interval measurements for each channel using the absolute TOA method, for the phantom with gelatine concentration of 7.5%.

Channel number	Shear wave speed (m/s)	SD (m/s)
1	1.86	±1.15
2	1.78	±1.00
3	1.59	±0.76
4	1.86	±0.91
Mean (± inter-group SD)	1.77±0.13	0.96
± overall SD	±0.86	

**Table 5:** The shear-wave speed using the 4-channel method for the phantom with a gelatine concentration of 7.5%.

Group number	Shear-wave speed (m/s)	R^2^
1	1.02	0.99
2	1.07	1
3	1.00	1
4	1.01	1
5	0.99	0.99
6	0.98	1
7	1.07	1
Mean (± inter-group SD)	1.02±0.04	1

**Table 6:** The shear-wave speed using the absolute multi-group method for the phantom with a gelatine concentration of 7.5%.

Channel number	Shear-wave speed (m/s)	R^2^
1	1.02	0.92
2	1.06	0.93
3	1.05	0.94
4	1.07	0.94
Mean (± inter-group SD)	1.05±0.02	0.93

**Fig 7:**
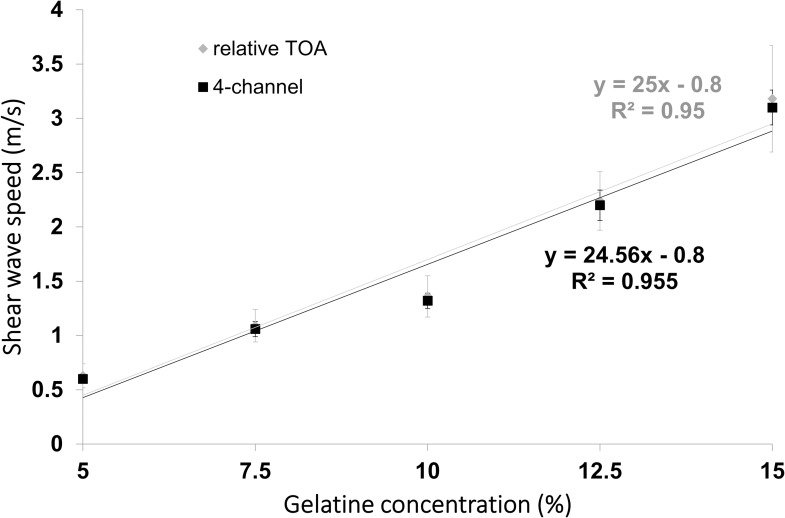
The shear-wave speed (with error bars indicating ±1 standard deviation) as a function of gelatine concentration for the relative 2-channel TOA and the 4-channel methods.

The trends of mean value as a function of gelatine concentration, and overall standard deviation for each estimate of shear- wave speed, are compared for the relative (2-channel) and 4-channel TOA methods in Fig 7. Also shown for each of the methods is a straight line fitted by the method of least squares, a chi-squared statistic which represents a measure of goodness of fit of the straight line to the data points and the correlation coefficient with p-value for the probability that there is no correlation.

Using the compressional rheometer the measured shear wave modulus was 9.9 ± 1 GPa and using a torsional rheometer the value was 9.8 ± 1.9 GPa. As shown in Fig 7 the shear-wave speed obtained by the 2-channel method was 3.18 ± 0.49 m/s and 3.10 ± 0.17 m/s by the 4-channel method. Taking ρ = 1 in Eq 1, we obtain 10.11 ± 2.26 m/s and 9.61 ± 0.75 m/s respectively.

## Discussion

From the above results a number of observations can be made with regard to the absolute and relative methods of shear wave calculation. As it is apparent from Table 4, the absolute method in our case is not able to provide the useful precision owing to the lack of a precise synchronization between signal initiation and data acquisition. This could be referred as a jitter. The origin of the jitter is the noise present throughout the entire trace, which translates into temporal jitter when triggering the signal initiation. With a typical range of ±0.1 V, i.e. 2.5% of the total width of the B-mode scan (data not shown), it seems unlikely that this level of noise is present on the actual signal that drives the mirror. Rather, its presence may be due to radiofrequency interference picked up due to insufficient shielding on the cable used to bring the signal from the circuit board of the scanner. It can be concluded therefore, that the jitter is not fundamental to the method, and that the situation could be improved by either i) better shielding, ii) the use of a simple resistor-capacitor smoothing circuit with a time constant that smooths the noise but preserves the rising voltage edge that represents the trigger, or iii) the use of an entirely different method of deriving a trigger. Solving jitter problem is especially important in the view of our goal of studying cellular medium which exhibits non-linear behaviour. The absolute TOA method will be applied to short segments in which a time delay will be calculated, therefore treating each segment linearly. Despite the stated above, absolute TOA method could still be useful when the size of the segment is such that it can include several groups as demonstrated in Table 6.

The relative method in general was shown to be more precise, for it is not influenced by the jitter. The error of this method, as shown in Table 3, is a standard deviation of three inter-channel results and does not represent the error of one channel measurement, because the latter can be obtained only by repeating the experiment. But if we assume the homogeneity of the gel, this definition of a standard deviation should be sufficient. Table 3 also shows that the overall 21 measurements contribute substantially more to the standard deviation (SD = 0.10) rather than inter-group differences (SD = 0.02). The first one is in close accordance with the inter-group differences (SD = 0.09), which therefore could be regarded as a main source of error. It should be noted that in contrast to the absolute method, in the relative method the compared waveforms are taken from different axial positions. But since there was no detectable difference in TOA from different channels, the generated wave should be regarded as propagating in lateral direction only.

The 4-channel method is the most precise among two relative and two absolute TOA methods, but the size of the segment is 0.552 mm in contrast to 0.184 mm of relative 2-channel method. In addition, lower standard deviation in the measurements of this method in comparison to 2-channel method as shown in Fig 7 proves that the method of plotting a graph through three points and obtaining the speed from the slope is better than averaging the speeds from three inter-channel calculations.

Although the results in Fig 7 confirm that for homogenous gels the speed of the shear waves increases in proportion with gelatine concentration, it is difficult to compare our quantitative results with those reported in the literature, because gelatine types vary in their strength (indicated by a bloom number) and each type has different mechanical properties. Moreover, each single result will depend not only on gelatine concentration, but also on the temperature during the experiment, the time of gelatine exposure to the air and some human effects, such as a thorough mixture during preparation. Qualitatively, however, with other conditions remain the same (in particular gel thickness and frequency), the stiffness increases with concentration. A linear relationship between the two was anticipated from the argument presented in the Materials and Methods section and was confirmed by the FEM results (Fig 5). We can conclude that for the range of shear moduli studied and the thickness of the layer, even within the thin layer, the assumption of a simple linear relationship between shear modulus and the transverse wave speed is justified. The dip in shear-wave speed at 10% gelatine concentration seen in Fig 7, relative to this linear model, therefore seems unlikely to be from systematic effects due to propagation in a thin layer, since the latter would predict curvature in the opposite direction to that observed.

The comparison between a direct and indirect shear modulus measurement has shown that the two are in a good agreement with each other, although we may expect a presence of guided shear wave at the given conditions. We expect that the good agreement between the two should exist also at lower gel concentrations, as for them the length of the propagating shear wave is shorter and therefore they will not ‘feel’ the boundaries of the medium.

A similar experimental arrangement was employed by Orescanin et al. that used a similar set-up to assess the stiffness of culture gels, but instead of OCT they used low frequency ultrasound [25]. Although the obtained shear-wave speed results correlate well with the chosen viscoelastic model, it lacks the required microscopic resolution, which potentially can be improved by using high-frequency ultrasound. Chen et al. introduced MR needle driven shear-wave elastography and showed good correlation with independent mechanical compression testing [26]. However a high resolution of MR elastography cannot compete with the robustness, speed and cost of the currently presented method. OCT was employed in measuring the speed of shear-wave propagation generated by acoustic radiation force [35]. The calculation of the speed was based on measuring the OCT phase. Phase measurement was also employed by Song et al., but their lateral resolution was not better than our 4-channel method [36].

The main limitation of the relative and the 4-channel methods is a constant size of a segment on which the stiffness is measured. The minimal length is 184 μm, whilst the maximal is about half a millimeter. Therefore, the initial aim of measuring the stiffness of cellular environment is feasible, given the fact that cellular cluster can be several hundred microns in diameter. Moreover, for heterogeneous samples the transition in stiffness will be gradual and not sharp. This gradual transition is directly related to the final resolution of the emerging elastic microscope and this feature is a topic of future study.

Further work is required to extend the method to non-homogenous gelatine phantoms, create three dimensional elastograms, determine suitability to biological experiments of interest as well as to establish a theory about the nature of the propagating waves. Typically, the waves that propagate in thin layers are Lamb waves which are transverse waves in bounded media. Another type of wave that propagates in the medium are Rayleigh waves, which are restricted to the surface of the gel. But since the gel layer is only 5 mm deep, Rayleigh waves penetrate into the depth and interact with the Lamb waves. This complicated nature of the shear waves in a bounded medium should be studied together with channel dependency, since each channel has its focus in different depth. A next step, for example, could be the use of the phase of the OCT backscatter signal [37], which should enable much greater sensitivity to small displacements.

## Conclusions

Quantitative OCE of small homogeneous samples has been accomplished using relatively simple modifications to a commercially available OCT system, without availability of phase or accurate synchronisation information.

The novel relative TOA and 4-channel methods provide better resolution than the implemented absolute method. Nevertheless, the spatial resolution is less that would be desired for cellular media studies. The repair of the jitter phenomenon will allow even better results and higher potential resolution of the device by using the absolute synchronisation method to make TOF measurements between shorter segments than 184 μm. The approach shows promise for its eventual application as a research tool in cancer cell biology.
